# RANKL/RANK: from bone loss to the prevention of breast cancer

**DOI:** 10.1098/rsob.160230

**Published:** 2016-11-23

**Authors:** Verena Sigl, Laundette P. Jones, Josef M. Penninger

**Affiliations:** 1 IMBA, Institute of Molecular Biotechnology of the Austrian Academy of Sciences, Dr Bohrgasse 3, 1030 Vienna, Austria; 2 School of Medicine, Department of Pharmacology, University of Maryland, Baltimore, MD 21201, USA

**Keywords:** breast cancer, RANK/RANKL, BRCA1

## Abstract

RANK and RANKL, a receptor ligand pair belonging to the tumour necrosis factor family, are the critical regulators of osteoclast development and bone metabolism. Besides their essential function in bone, RANK and RANKL have also been identified as the key factors for the formation of a lactating mammary gland in pregnancy. Mechanistically, RANK and RANKL link the sex hormone progesterone with stem cell expansion and proliferation of mammary epithelial cells. Based on their normal physiology, RANKL/RANK control the onset of hormone-induced breast cancer through the expansion of mammary progenitor cells. Recently, we and others were able to show that RANK and RANKL are also critical regulators of *BRCA1*-mutation-driven breast cancer. Currently, the preventive strategy for *BRCA1*-mutation carriers includes preventive mastectomy, associated with wide-ranging risks and psychosocial effects. The search for an alternative non-invasive prevention strategy is therefore of paramount importance. As our work strongly implicates RANK and RANKL as key molecules involved in the initiation of BRCA1-associated breast cancer, we propose that anti-RANKL therapy could be a feasible preventive strategy for women carrying *BRCA1* mutations, and by extension to other women with high risk of breast cancer.

## 1. Introduction

**Figure 1.**
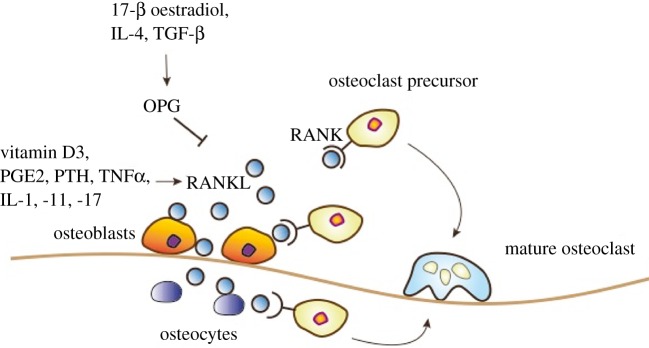
RANK/RANKL-mediated osteoclastogenesis. RANKL expression by osteocytes and osteoblasts is induced by vitamin D3, prostaglandin E2 (PGE2), parathyroid hormone (PTH) and several cytokines including tumour necrosis factor α (TNFα) and interleukin (IL)-1, -11 and -17. Interaction of membrane bound or soluble RANKL with RANK-expressing osteoclast precursor cells induces their differentiation and activation into mature osteoclasts. Expression of the decoy receptor osteoprotegerin (OPG) is induced by 17-β oestradiol, IL-4 or transforming growth factor β (TGF-β). OPG interferes with RANK/RANKL interaction thereby inhibition bone degradation.

Bone is a tissue that is continuously being rebuilt and remodelled. Osteoblasts are bone building cells that deposit new bone tissue, whereas osteoclasts are bone resorbing cells that are responsible for breaking down bone. Through a delicate balance between these two cell types, the skeleton is subjected to continuous change [1]. RANKL (TNFSF11) and RANK (TNFRSF11A), a receptor ligand pair of the tumour necrosis factor (TNF) receptor superfamily, have emerged as the key molecular pathways in bone physiology being essential for osteoclast development [2,3] (figure 1). RANKL, either present in a soluble or membrane bound form, can be induced by various stimuli, including the sex hormone progesterone [4]. Expression of the RANKL-decoy receptor osteoprotegerin (OPG) is strongly dependent on oestrogen, thereby linking oestrogen to bone turnover [5,6]. In postmenopausal women, the loss of oestrogen leads to reduced OPG and thus a relative rise of RANKL activity, ultimately leading to enhanced bone turnover and osteoporosis [7,8]. Osteoporosis is a state of decreased bone mass with increased risks of fractures resulting in hospitalization and is one of the most frequent causes of death in the elderly. In women with osteoporosis, elevated RANKL plasma levels as well as increased plasma OPG levels and a higher OPG/RANKL ratio can be detected [9]. The elevation of plasma OPG levels could be explained as a compensatory mechanism for increased RANKL levels. The fully human monoclonal RANKL-blocking antibody denosumab has been developed and approved for the treatment of osteoporosis and skeletal related events in cancer [10], already benefiting tens of thousands of patients. Besides the crucial function in osteoclastogenesis, RANK and RANKL have been implicated in various other physiological processes including immunotolerance, organogenesis of the immune system [2,3], osteoimmunology [11], hair growth [12] or thermoregulation in the central nervous system [13]. For example, bone loss that frequently occurs under inflammatory conditions such as rheumatoid arthritis can be, in part, explained by RANKL-expressing activated T cells [14]. RANK and RANKL are also essential for lymph node organogenesis, because *Rank*^−/–^ and *Rankl*^−/–^ mice completely lack lymph nodes [2,3]. Furthermore, RANK and RANKL have been shown to positively regulate the survival of dendritic cells and T cell functions [15]. RANK and RANKL are also implicated in the establishment of central immunotolerance by inducing the development of CD80^+^Aire^+^ medullary thymic epithelial cells and by regulating regulatory T cell (Treg) homeostasis [16]. Of note, tumour infiltrating RANKL expressing Tregs have further been associated with the promotion of metastasis of ERB-B2/NEU-positive mammary cancer cells to the lung [17]. Recently, a second RANKL receptor, LGR4, has been reported that exerts opposite effects of RANK during osteoclast differentiation [18]. LGR4 (leucine-rich repeat-containing G-protein-coupled receptor 4) is implicated in the regulation of multiple developmental pathways and signals either through classical G-protein signalling or through Wnt signalling [18].

One of the most surprising functions of RANK and RANKL is their essential role in mammary gland development during pregnancy [4]. *Rank* and *rankl* knockout mice display a complete block in the formation of a lactating mammary gland during pregnancy, resulting in a lactation defect and the subsequent indirect death of newborn pups, because they cannot be nurtured by their *rank* or *rankl* deficient mothers; the pups can, however, survive with a wild-type foster mother, though homozygous knockout mice exhibit the described phenotypic alterations [4]. Mammary gland development during pregnancy is mainly induced by progesterone, prolactin and parathyroid hormone related peptide (PTHrP) [19]. In particular, progesterone is crucial for proliferation of mammary epithelial cells that then differentiate into milk-secreting acini. Mechanistically, progesterone induces RANKL expression in hormone-receptor positive progenitor cells resulting in proliferation of neighbouring RANK-expressing, hormone receptor negative mammary epithelial progenitor cells, a mechanism that appears to be active in every oestrous cycle and is critical to expand the epithelial mammary tree during pregnancy [20,21].

## 2. RANK/RANKL couple sex hormones to mammary stem cells

The mammary gland is organized into two main cell types, namely the luminal and myoepithelial lineage. Luminal cells can be subdivided into ductal and alveolar cells and are mainly responsible for the mammary secretion of fluids and nutrients. Myoepithelial lineage cells are also referred to as basal cells because they are located adjacent to the basement membrane and can exert contractile functions, thereby guiding the milk through the epithelial tree [22]. While it was thought for a long time that mammary progenitors reside in a quiescent stem cell niche, it has become clear that mammary progenitor cells undergo proliferation and differentiation during each oestrus cycle [20,21]. Moreover, mammary stem cell numbers change during the course of each oestrus cycle, during pregnancy as well as during ageing, thereby allowing the mammary gland to adapt to altered physiological states [20,21].

Mammary stem cells are highly enriched in a basal epithelial population, self-renew and are able to generate all mature cell types of the mammary gland; a single mammary stem cell is able to reconstitute a fully functional mammary gland, which can even undergo further development and milk production during pregnancy [22,23]. Although the mammary stem cell enriched subsets in mouse and human lack expression of oestrogen and progesterone receptors, these cells are highly responsive to steroid sex hormones. During the oestrous phase of each cycle, progesterone induces the expansion of mammary stem cells through paracrine mechanisms. Similarly, administration of exogenous oestrogen and progesterone also increases of the numbers and repopulation capacities of mammary stem cells. By contrast, ovariectomy or treatment with aromatase inhibitors significantly reduces mammary stem cell activity [20,21]. During pregnancy a dramatic increase in mammary stem cell numbers as well as enhanced repopulation capacity can be observed [20,21]. This might explain the transient increase of breast cancer risk during pregnancy. However, pregnancies in younger women reduce the risk of developing breast cancer later in life [24]. Mouse studies have shown that the stem cell pool is significantly diminished in parous mice, thereby possibly explaining the reduced breast cancer risk after an early pregnancy [25].

**Figure 2.**
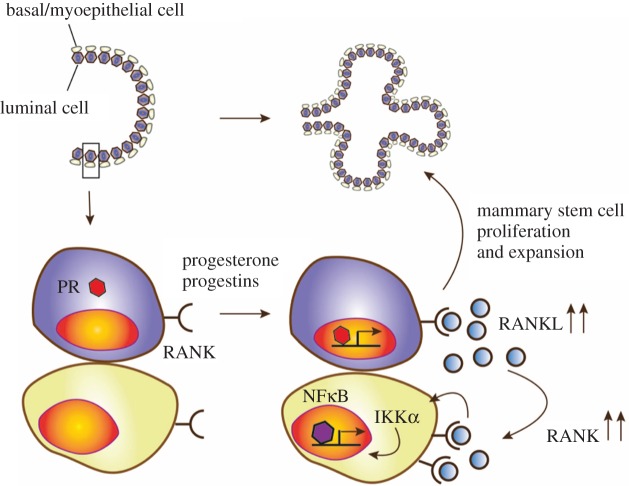
RANK/RANKL-mediated mammary stem cell expansion. RANK is constitutively expressed on the surface of basal and luminal mammary epithelial cells. Progesterone, progestins and prolactin (and possibly other yet unknown factors) induce RANKL expression in progesterone receptor-positive basal mammary cells including mammary stem cells. Binding of RANKL to RANK on luminal epithelial cells takes place in an autocrine manner, whereas RANKL binding to RANK on basal mammary epithelial cells depends on a paracrine activation loop. RANK/RANKL interaction on basal mammary epithelial cells induced further upregulation of RANK and the induction of the IKKα–NFκB–Cyclin D1 signalling axis resulting in proliferation and expansion of mammary stem cells.

**Figure 3.**
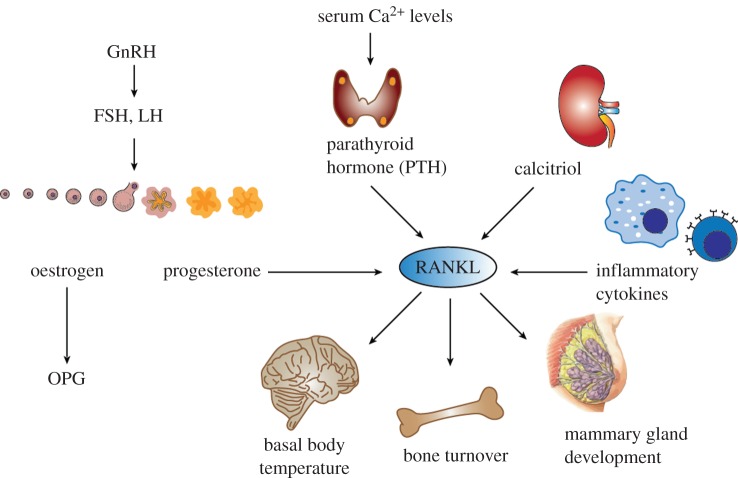
Mediators regulating RANKL expression. Progesterone, which is secreted during the luteal phase of the menstrual cycle as well as during pregnancy, is a strong inducer of RANKL expression. By contrast, oestrogen, which is the dominating hormone during the follicular phase of the menstrual cycle, induces the expression of the decoy receptor OPG. RANKL expression is also induced by parathyroid hormone, a central regulator of calcium homeostasis. Further, calcitriol (vitamin D3) has been shown to induce RANKL expression. Moreover, inflammatory cytokines secreted by immune cells are able to induce RANKL expression. Depending on the physiological context, RANKL is upregulated in different target tissues, including the central nervous system, bone tissue and the mammary gland to exert its biological functions.

One key finding was that RANK and RANKL are essential for the dynamic cycling of the mammary stem cell pool during the normal oestrous cycle. Mechanistically, progesterone induces progesterone receptor (PR)-positive mammary epithelial cells to secrete RANKL. RANKL in turn acts in a paracrine fashion on adjacent hormone receptor negative, RANK-expressing mammary progenitor cells and induces their expansion [20,21] (figure 2). Moreover, RANKL can act in an autocrine manner on RANK positive luminal cells. Of note, the mammary gland in RANK and RANKL knockout mice develops normally during puberty, which can be explained by these changes in adolescence being primarily induced by oestrogen [4]. Importantly, as first described by our group, RANKL/RANK are absolutely required to drive mammary progenitors into the cell cycle during pregnancy, primarily induced by progesterone; this cell expansion is required for the formation of a lactating mammary gland [4]. Moreover, it has been shown that RANK stimulation induces R-spondin, thereby coupling sex hormone regulated RANKL/RANK to the Wnt pathway in mammary progenitor cells [26]. Interestingly, it has been shown that the recently identified alternative RANKL receptor LGR4 is also critically involved in mammary gland development and mammary stem cell biology. Mice devoid of LGR4 display a delay in ductal development, a decreased number of terminal end buds and decreased side-branching of the epithelial tree. Moreover, the mammary stem cell repopulating capacity is severely impaired in *Lgr4* knockout mice [27]. Thus, it seems possible that LGR4 is not only involved in RANK/RANKL-mediated osteoclastogenesis, but also plays a role in RANK/RANKL-dependent mammary gland development and mammary stem cell regulation. The regulation of proliferation of mammary progenitors by the RANK/RANKL pathway appears to hold true in all mammals, at least to the extent this has been experimentally addressed [26]. Thus, RANK and RANKL are the essential effector molecules of progesterone to drive mammary progenitor cells into the cell cycle during the progesterone phase of the oestrous cycle and during pregnancy. Figure 3 depicts the hormone-dependent functions of RANK and RANKL in various organ systems.

## 3. The role of RANK and RANKL in breast cancer

Breast cancer is the most common female cancer and affects 1 in 8 women during their lifetime. Risk factors for breast cancer include exposure to environmental factors such as smoking, synthetic sex steroid hormones or genetic predisposition. In 2003, the Million Women Study and the Women's Health Initiative Study reported a significant increase in breast cancer risk in women using oestrogen plus progesterone hormone replacement therapy (combined HRT), compared with women using oestrogen only HRT [28,29]. These two studies provided strong population-based evidence that progesterone is a crucial factor for increased breast cancer risk in women. Ten years later, an extended post-intervention follow up study of the Women's Health Initiative Study did confirm the initial finding that the use of combined HRT markedly increases the risk of developing breast cancer [30].

Because progesterone is a prominent trigger of RANKL expression (while oestrogen regulates the molecular decoy receptor OPG), which in turn induces mammary progenitor cells to proliferate, the idea arose that RANK and RANKL might also be involved in pathologic changes of the breast tissue, namely sex hormone-driven mammary cancer [4,20,21]. Indeed, we were able to show that RANK and RANKL are critical in the development of hormone-induced breast cancer in mice [31,32]. Subcutaneous implantation of the synthetic progesterone medroxyprogesterone acetate (MPA) into female mice triggered massive upregulation of *Rankl* mRNA in mammary epithelial cells, resulting in proliferation of mammary epithelial progenitor cells [31]. To study the onset of mammary tumours in mice, mammary epithelial cell-specific *Rank* knockout mice were generated using the MMTV-Cre deleter line (Rank*^ΔMMTV^*). Mammary tumours were then induced using the carcinogenic agent 7,14-dimethylbenz(α)anthracene (DMBA) in combination with MPA. Intriguingly, Rank*^ΔMMTV^* mice displayed a delayed onset and incidence of mammary tumours compared with wild-type littermate controls [31]. Previously, it has been shown that IKKα acts as a main RANK downstream signalling pathway in mammary epithelial cells [33,34]. MMTV-driven deletion of *Ikkα* (IKK*^ΔMMTV^*) in mammary epithelial cells also resulted in a delayed onset of mammary tumours following DMBA/MPA treatment, suggesting that RANK/RANKL signals through IKKα in progestin-driven mammary cancer [31]. Mechanistically, RANK/RANKL confer resistance to γ-irradiation-induced cell death in mammary epithelial cells, change cell adhesion and regulate self-renewal capacity of tumour stem cells, all of which might contribute to how RANKL/RANK drive mammary cancer development [31]. At the same time, another group reported that treatment of mice using a selective pharmacological RANKL inhibitor, RANK-Fc, almost completely blocked the occurrence of DMBA/MPA-induced mammary tumours in wild-type mice [32]. Thus, besides being critical regulators of mammary gland development during pregnancy and mammary stem cell numbers in the oestrous cycle, RANK and RANKL control the onset of hormone-induced mammary cancer.

## 4. *BRCA1* mutations in breast cancer

Most breast cancers develop in women during their sixth decade of life and occur sporadically. Approximately 5–10% of breast cancers are caused by inherited mutations [35]. For a significant part of inherited breast cancers, a specific underlying mutation or a distinct inheritance pattern cannot be identified; these cases are referred to as familial breast and ovarian cancer syndrome and are characterized by the frequent occurrence of breast and ovarian cancer within one family line. The aetiology is multifactorial and based on several mutations that predispose to cancer [36,37]. Besides the non-identifiable mutations, there are a few specific germline mutations that predispose to breast cancer. These specific genetic changes include for example germline mutations in *STK11* causing Peutz–Jeghers syndrome, *ATM* causing ataxia teleangiectasia or Louis-Bar syndrome, *PTEN* causing Cowden syndrome, or *p53* causing Li Fraumeni syndrome as well as germline mutations in *CHEK-2*. However, besides increasing risk of developing breast cancer and other types of cancers, germline mutations in some of these genes frequently cause developmental defects that limit life expectancy [38].

Within the group of inherited breast cancer cases, the vast majority is caused by mutations in the tumour suppressor genes *BRCA1* and *BRCA2*. *BRCA1-*mutation carriers have a lifetime risk of up to 85% of developing breast cancer and about 45% of developing ovarian cancer [36,39]. Germline mutations in the *BRCA2* gene increase the lifetime risk of developing breast cancer by up to 66% and ovarian cancer by approximately 12%. *BRCA2* mutations also predispose to cancers of the male breast, pancreas, prostate and other organs [37]. Starting at the age of 25, the risk of developing breast cancer rises continuously in *BRCA1-*mutation carriers. The highest breast cancer incidence in these patients occurs in premenopausal women during their fourth decade of life. The risk of developing ovarian cancer in *BRCA1-*mutation carriers is minor before the age of 40 but then also shows a continuous rise [36,39]. *BRCA1*-mutant tumours generally show a considerable molecular, histological and clinical heterogeneity. Breast cancers particularly arising in *BRCA1*-mutation carriers often exhibit basal-like characteristics, defined by the expression of genes specific to the basal mammary myoepithelial cells. Moreover, *BRCA1*-mutated breast cancers are frequently triple negative and by definition lack the expression of oestrogen receptor (ER), PR and amplification of the ERBB2 oncogene [36,40,41]. By contrast, 77% of breast tumours arising in *BRCA2* mutation carriers are ER-positive and only 16% are found to be triple negative, paralleling the breast cancer subtypes seen in the general population [42].

*BRCA1* is located on the long arm of chromosome 17 (17q21) consisting of 24 exons, of which 22 are coding for a 1863 amino acid protein [43]. *BRCA2* is located on chromosome 13q12.3 and consists of 27 exons, which code for a 3418 amino acid protein, one of the biggest polypeptides of the human proteome [44]. Both proteins interact with multiple genes that are involved in DNA repair, e.g. with Rad51 which has a key function in DNA double strand break repair. Loss of *BRCA1* and *BRCA2* therefore leads to defective repair of damaged DNA and hence increases the susceptibly to genotoxins as well as spontaneous chromosomal aberrations. The increased cancer susceptibility in *BRCA1* and *BRCA2* mutation carriers therefore can—to a large extent—be explained by chromosomal instability [45,46]. In addition, *BRCA1* and *BRCA2* have been shown to play a role in telomere maintenance and cell cycle progression [47,48]. Genetic inactivation of either *BRCA1* or *BRCA2* in mice indeed results in embryonic lethality due to reduced cellular proliferation [49].

Despite its ubiquitous expression, it is thought that *BRCA1* mutations increase the susceptibility of developing breast cancer through tissue-specific functions beyond the maintenance of chromosomal integrity. Besides its function in DNA repair, BRCA1 has been shown to be involved in the transcriptional regulation of ER and PR expression [50–52]. Moreover, BRCA1 has been shown to regulate genes that are critical for normal differentiation of luminal mammary epithelial cells [53]. Mammary gland specific deletion of *BRCA1* results in the accumulation of mammary epithelial progenitors that display defective differentiation, elevated c-Kit expression and growth factor independent growth *in vitro*. BRCA1 has also been identified as a potential positive regulator of mammary stem cells because deletion of *BRCA1* results in a reduced repopulating frequency *in vivo* [53]. Of note, p53, which is often mutated in women with *BRCA1*-associated breast cancer, is a known negative regulator of mammary stem cells and deletion of p53 results in the expansion of stem cells *in vitro* and *in vivo* [54,55]. Thus, the specific functions of BRCA1 in the mammary gland and steroid hormone signalling might possibly explain the tissue specificity of tumours arising in *BRCA1*-mutation carriers.

The discovery of BRCA1 and BRCA2 made it for the first time possible to offer genetic testing to determine breast cancer risk [43]. The underlying carrier rate for *BRCA1* and *BRCA2* mutations is assumed to be one in 399 [56]. However, the distribution of *BRCA* mutations in the general population varies according to the ethno-cultural origin. For instance, in the US population in general, it is estimated that between 1 out of 345 to 1 out of 1000 individuals carry a *BRCA* mutation, compared with approximately 1 in 40 individuals of Ashkenazi Jewish descent [57]. The clinical consequences of a confirmed *BRCA1* or *BRCA2* mutation include intensive screening for early tumour detection. *BRCA1-*mutation carriers mostly profit from bilateral mastectomy and prophylactic salpingo-oophorectomy [36]. However, the decision on these preventive options shows large variations by the country of residence [58]. In addition, breast and ovary removal is often associated which wide-ranging risks and psychosocial effects [36]. The search for an alternative non-invasive prevention strategy is therefore of paramount importance for many women carrying *BRCA* mutations, and by extension for all other women with increased risk of breast cancer.

## 5. RANK and RANKL link female sex hormones to *BRCA1* mutation-induced breast cancer

Numerous exogenous and endogenous factors are known to elevate the risk of developing breast cancer. Among these risk factors, the exposure to endogenous and exogenous sex hormones plays an important role in the development of breast cancer. For example, early menarche and late menopause correlates with an increased risk of developing breast cancer [59]. By contrast, early pregnancy and lactation decrease the risk of developing breast cancer [24,60]. Interestingly, epidemiological studies suggest that early pregnancies do not confer reduced breast cancer risk in women carrying a *BRCA1* mutation, but might even elevate cancer incidence [61]. Consequently, several studies have linked *BRCA1* mutation-mediated tumourigenesis to female sex hormones. *In vitro* studies have shown that BRCA1 interacts with oestrogen and progesterone receptors, inhibiting their transcriptional activity, and stimulation of *Brca1*-mammary gland specific knockout mice with exogenous progesterone resulted in increased proliferation of mammary epithelial cells [62]. Animal studies have also shown that *Brca1* deletion specifically in ovarian granulosa cells prolongs the pro-oestrous phase in mice, which corresponds to the follicular phase of the human menstrual cycle [63]. A direct effect of progesterone on *Brca1*-associated breast cancer was demonstrated in studies on *Brca1;p53* deficient mice; administration of the progesterone antagonist mifepristone markedly blocked tumour development in this mouse model [64]. Importantly, prophylactic salpingo-oophorectomy significantly and substantially reduces the risk of breast cancer development in *BRCA1*-mutation carriers (by 75%) [36,39]. Thus, in mouse studies and in humans, sex hormones play a significant role in the pathogenesis of *BRCA1-*mutated breast cancer.

The fact that breast cancer development in *BRCA1-*mutation carriers is crucially affected by female sex hormones led to the question whether RANK/RANKL, which are key molecules in the mammary gland downstream of steroid hormones, have a role in the aetiology of *BRCA1* mutation-driven breast cancer. A first hint that RANK/RANKL might indeed be involved in the pathogenesis of *BRCA1*-mutated breast cancer came from studies of sex hormone titres in women with a *BRCA1* mutation. Women carrying a germline *BRCA1* mutation have higher progesterone titres as well as higher oestrogen titres during the luteal phase compared with their sisters not carrying the mutation [65]. Furthermore, it was shown, that serum levels of RANKL and its decoy receptor OPG are deregulated in *BRCA1*-mutation carriers, lower OPG serum levels being associated with an increased risk of breast cancer [66]. However, these were all correlation studies and serum levels of RANKL and OPG in most cases do not reflect the underlying physiological or pathological conditions, e.g. RANKL or OPG serum levels do not correspond to osteoporosis. For instance, low serum RANKL levels are associated with a 10-fold higher risk of non-traumatic fractures in postmenopausal women [67]. Moreover, increased OPG is associated with enhanced bone loss in postmenopausal women not on hormone replacement therapy [68] and increased OPG levels have been observed in patients with bone metastasis [69]. The mechanisms of these serum changes need to be further investigated and might reflect compensatory mechanisms and/or redistribution/sequestration of RANKL/OPG within different body compartments.

Recently, we provided direct genetic evidence for the role of RANK/RANKL in *Brca1* mutation-driven breast cancer [70]. At four months of age mammary glands of mice carrying a *Brca1;p53* mutation using the Cre-deleter line K5Cre showed excessive proliferation and malignancy. Intriguingly, genetic deletion of *Rank* in these mice significantly reduced proliferation and almost completely abrogated the occurrence of malignancy. Moreover, in a second genetic model, whereas tumour incidence in *Brca1;p53;Wap^C^Cre* mice was 100%, 25% of mice with concomitant *Rank* deletion remained tumour free throughout their whole lifetime. Most importantly, we were also able to experimentally show that RANKL blockade could be used as a preventive strategy in *BRCA1*-mutation carriers: RANKL blockade in mice carrying a *Bcra1* mutation markedly abrogated the occurrence of pre-neoplastic lesions, and after 1 year of treatment only 1 out 13 *Brca1*-mutant mice showed mammary epithelial neoplasia whereas nearly all untreated control mice developed mammary tumours. Mechanistically, we could show that *Brca1* mutations result in an increase of mammary progenitor cell numbers that is dependent on RANK/RANKL. Most importantly, colony-forming capacity of human mammary epithelial progenitor cells from heterozygous *BRCA1*-mutation carriers can be significantly decreased by treatment with denosumab. Furthermore, we found high RANK expression in pre-malignant lesions and breast cancer samples from patients carrying a *BRCA1* mutation. Finally, having obtained access to data from the Collaborative Oncological Gene-environment Study (iCOGS) that included approximately 15 200 *BRCA1* and approximately 8200 *BRCA2* mutation carriers, we found six SNPs in the *TNFRSF11A* locus (*TNFRSF11A* codes for RANK) that were significantly associated with breast cancer risk in the overall series of *BRCA1-*mutation carriers [70]. Thus, common variations in *TNFRSF11A* modify the risk of developing breast cancer in *BRCA1-*mutation carriers, data that should be replicated in additional and larger datasets.

**Figure 4.**
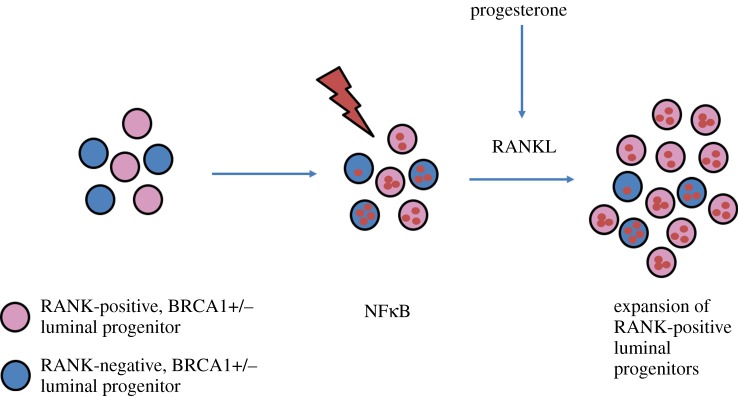
RANK/RANKL-mediated proliferation of BRCA1+/− mammary progenitor cells. RANK is constitutively expressed on the surface of a subset of luminal mammary epithelial progenitor cells. Mammary cells carrying a BRCA1+/− mutation accumulate DNA damage due to an increased susceptibility to genotoxic stress. Increased DNA damage leads to the constitutive activation of NFκB in luminal progenitor cells. Progesterone-induced RANKL expression triggers the expansion of RANK-positive mammary luminal progenitor cells contributing to the development of mammary tumours.

Our results were confirmed by other recently published studies, reporting that proliferation of organoids derived from breast biopsies of *BRCA1*-mutation carriers was markedly reduced when RANKL was blocked with the RANKL-blocking antibody denosumab. Furthermore, these authors also found a significant RANKL-dependent reduction of tumour incidence in a mouse model of transplanted mammary tumours [71]. Moreover, in another study, nuclear factor kappaB (NFκB), a well-known downstream target of RANK, was shown to be persistently activated by DNA damage in *BRCA1*-deficient mammary progenitors and *in vivo* inhibition of NFκB prevented hormone-independent colony formation of luminal progenitor cells [72]. Figure 4 depicts the current understanding of RANKL/RANKL function in *BRCA1*-mutated breast cancer. Taken together, results from different laboratories strongly indicate that RANKL/RANK act on murine as well as human mammary progenitor cells and that RANKL/RANK are critical regulators for the initiation as well as progression of *BRCA1* mutation-driven mammary cancer.

## 6. Denosumab: a way to prevent breast cancer?

It is well recognized that particular environmental factors can modify the risk of developing breast cancer in the general population. Moreover, screening methods are commonly used for the detection of breast cancers at an early stage. Many women have certainly profited from these screening strategies, because early detection of breast cancer is associated with better prognosis [73]. However, besides careful monitoring and life planning followed by mastectomy—in terms of *BRCA1* carriers—there is no current drug that is suitable for the prevention of breast cancer. Based on all available data in cancer models and its physiological role in mammary epithelial proliferation in—as far as we know—every pregnant mammal, RANKL blockade could be a feasible option for the prevention of breast cancer in *BRCA1-*mutation carriers.

Denosumab, a monoclonal fully human RANKL-blocking antibody was initially developed for the treatment of osteoporosis. After successful completion of clinical trials, denosumab was approved for the treatment of postmenopausal osteoporosis and the prevention of skeletal related events in patients with solid tumours [10]. To date, already thousands of patients worldwide have received denosumab. Most importantly, the administration of denosumab is well tolerated. The most severe side effects, when given at high doses in cancer patients, include necrosis of the jaw bone and hypercalcaemia. However, proper dental hygiene and dental restoration prior to the treatment as well as regular laboratory controls for calcium levels can help to circumvent these complications [10]. By contrast, the therapeutic use of the anti-progesterone mifepristone (RU-486), which in animal experiments also potently prevents tumour development in *Brca1;p53;Wap^C^Cre* mice [74], is limited due to toxicity.

As denosumab has been approved for use in humans, the drug is immediately available to test its efficacy on breast cancer prevention in *BRCA1-*mutation carriers in clinical trials. Besides *BRCA1* (and *BRCA2*) mutation carriers, also other women with increased risk of breast cancer could benefit from such a prevention strategy. In 2010, we first showed that RANK and RANKL control hormone-induced breast cancer [31,32]. Moreover, in a recent clinical study, initially designed to test the effect of denosumab on bone health in breast cancer patients receiving endocrine therapy (aromatase inhibitors), the recurrence of breast cancer was markedly reduced in the denosumab-treated group compared with the control group. Of note, this clinical trial was prematurely terminated because the strong positive bone protective effects of RANKL inhibition rendered it unethical to withhold denosumab from the untreated group. The same trial also showed that, based on 11 000 patient years, low dose of denosumab (two injections per year, as used for osteoporosis) are perfectly safe [75]. These results are very encouraging in terms of efficacy and safety and support the notion that RANKL blockade could be used as a preventative strategy to reduce the incidence of breast cancer.

## 7. Conclusion

Almost 20 years ago, RANK and RANKL were identified as new members of the tumours necrosis factor/TNF-receptor family of proteins. Initially, RANK and RANKL were found to be the key regulators of bone metabolism controlling osteoclast differentiation and activation. Now many years have passed and many more functions of RANK and RANKL have been identified. Some of these functions might have been expected, however some of them were a surprise. These functions of RANK and RANKL range from bone turnover to immune regulation, development of secondary lymphoid organs, fever regulation, regulation of bone metastases, and, importantly for this review, the establishment of a lactating mammary gland and the development of hormone- and *Brca1* mutation-induced mammary tumours.

In the mammary gland, RANKL is induced by the sex hormone progesterone and acts in a paracrine fashion on hormone receptor-negative RANK-expressing epithelial cells, inducing expansion of mammary progenitor cells. Most recently, we and others provided genetic and functional evidence for the critical function of RANK/RANKL in the development of familial *BRCA1*-mutated breast cancer. Thereby, RANK and RANKL control the expansion of RANK-positive luminal progenitor cells, eventually, e.g. under conditions of increased DNA damage, leading to the development of breast cancer. Moreover, the safety of pharmacological RANKL inhibition has been proven in thousands of women that have received the RANKL-blocking antibody denosumab up to date. Thus, RANKL inhibition using an approved drug might be a feasible strategy to, for the first time, prevent breast cancer in *BRCA1*-mutation carriers and possibly in other women at high risk for developing breast cancer. The next step will be to prove the efficacy of denosumab in *BRCA1*-mutation carriers in careful phase III clinical trials.
